# Drug use among medical students in São Paulo, Brazil: a cross-sectional study during the coronavirus disease 2019 pandemic

**DOI:** 10.1590/1516-3180.2022.0493.R1.150623

**Published:** 2023-09-08

**Authors:** Pedro Lemos-Santos, Lukas Blumrich, Jordi Blanes Debia, João Mauricio Castaldelli-Maia, Paulo Jeng Chian Suen, André Malbergier

**Affiliations:** I Undergraduate Student, Faculdade de Medicina da Universidade de São Paulo (FMUSP), São Paulo (SP), Brazil.; II Undergraduate Student, Faculdade de Medicina da Universidade de São Paulo (FMUSP), São Paulo (SP), Brazil; Doctoral Student, Department of Pediatrics, Faculdade de Medicina da Universidade de São Paulo (FMUSP), São Paulo (SP), Brazil.; Faculdade de Medicina da Universidade de São Paulo, Department of Pediatrics, São Paulo, SP, Brazil; III Undergraduate Student, Faculdade de Medicina da Universidade de São Paulo (FMUSP), São Paulo (SP), Brazil.; IV PhD, Postgraduate Sponsor, Department of Psychiatry, Faculdade de Medicina da Universidade de São Paulo (FMUSP), São Paulo (SP), Brazil; Assistant (Aux.) Professor, Department of Neuroscience, Centro Universitário Faculdade de Medicina do ABC (FMABC), Santo André (SP), Brazil.; Centro Universitário Faculdade de Medicina do ABC, Department of Neuroscience, Santo André, SP, Brazil; V Undergraduate Student, Faculdade de Medicina da Universidade de São Paulo (FMUSP), São Paulo (SP), Brazil; Doctoral Student, Department of Psychiatry, Faculdade de Medicina da Universidade de São Paulo (FMUSP), São Paulo (SP), Brazil.; Faculdade de Medicina da Universidade de São Paulo, Department of Psychiatry, São Paulo, SP, Brazil; VI PhD, General Coordinator, Interdisciplinary Group of Studies on Alcohol and Drugs (GREA), Institute of Psychiatry (IPq), Hospital das Clínicas da Universidade de São Paulo (HCFMUSP), São Paulo (SP), Brazil; Collaborating Professor, Department of Psychiatry, Faculdade de Medicina da Universidade de São Paulo (FMUSP), São Paulo (SP), Brazil.; Faculdade de Medicina da Universidade de São Paulo, Department of Psychiatry, São Paulo, SP, Brazil

**Keywords:** Students, medical, Prevalence, COVID-19, Mental health, Education, medical, Substance use, Psychoactive substances, University student drinking

## Abstract

**BACKGROUND:**

Medical students demonstrate higher rates of substance use than other university students and the general population. The challenges imposed by the coronavirus disease 2019 (COVID-19) pandemic raised significant concerns about mental health and substance use.

**OBJECTIVES:**

Assess the current prevalence of substance use among medical students at the University of São Paulo and evaluate the impact of the COVID-19 pandemic on drug consumption.

**DESIGN AND SETTING:**

A cross-sectional study was conducted on 275 medical students from the University of São Paulo Medical School (São Paulo, Brazil) in August 2020.

**METHODS:**

Substance use (lifetime, previous 12 months, and frequency of use before and during the COVID-19 pandemic) and socioeconomic data were assessed using an online self-administered questionnaire. Symptoms of depression were assessed using the Patient Health Questionnaire-9.

**RESULTS:**

Alcohol was the most consumed substance in their lifetime (95.6%), followed by illicit drugs (61.1%), marijuana (60%), and tobacco (57.5%). The most commonly consumed substances in the previous year were alcohol (82.9%), illicit drugs (44.7%), marijuana (42.5%), and tobacco (36%). Students in the first two academic years consumed fewer substances than those from higher years. There was a decreasing trend in the prevalence of most substances used after the COVID-19 pandemic among sporadic users. However, frequent users maintained their drug use patterns.

**CONCLUSION:**

The prevalence of substance use was high in this population and increased from the basic to the clinical cycle. The COVID-19 pandemic may have affected the frequency of drug use and prevalence estimates.

## INTRODUCTION

Substance use is a complex phenomenon including health, socioeconomic, and criminal dimensions that occur globally and affect all age ranges. According to the United Nations Office on Drugs and Crime, global substance use is rising, increasing from 4.8% of the global population aged 15–64 in 2009 to 5.5% in 2019.^1^ Young adults, especially university students, are among the most vulnerable groups associated with risky behaviors, including substance use.^2^ University student of alcohol and illicit drug use are reportedly higher than those from the general population.^3^

Among the university population, medical students use more alcohol and other drugs than non-medical ones.^4^ Additionally, stress, competitiveness, high pressure to perform, lack of sleep, changes in social support (parental and companionship) during university, a tendency towards unhealthy diets, and little exercise are some of the issues related to psychological pain and drug use among this population.^5^

Constant drug-related medical and social changes can alter the prevalence of drug use among medical students, highlighting the need to monitor this behavior. The liberalization of cannabis in many countries,^6^ using psychoactive substances (especially psychedelics) to treat psychiatric disorders,^7–10^ and using psychostimulants (especially methylphenidate) to improve academic performance,^11^ are some issues potentially associated with increasing substance use in this population. Moreover, drug consumption can begin in medical school and continue throughout professional life,^12^ impacting physicians quality of life and exposing patients to potential risk.^13^ Substance use disorders are likely to affect 8–15% of all physicians.^14^ Since this behavior seems to begin in the university setting, assessment and management of medical students' substance use may be of great importance.

Additionally, in 2020, the challenges imposed by the coronavirus disease 2019 (COVID-19) pandemic, especially self-quarantine and social distancing, led to significant concerns about mental health^15^ and substance use. Quarantine was shown to affect the consumption of alcohol among students^16^ as well as tobacco and other illicit drugs among the general population.^17–19^

All the issues mentioned above indicate the need for conducting studies on substance use among medical students. Therefore, this study aimed to evaluate the current prevalence of alcohol, tobacco, and illicit drug use among medical students and assess how the COVID-19 pandemic may have affected this behavior.

## METHODS

### Study design

A cross-sectional study was conducted among medical students at the Faculdade de Medicina da Universidade de São Paulo (FMUSP) in São Paulo, Brazil, in August 2020. Online questionnaires were sent to students institutional e-mail addresses. Data were collected through online questionnaires administered at the Research Electronic Data Capture platform.^20^

The study protocol was approved by the Ethical Committee of the Hospital das Clínicas of the FMUSP (approval number: 33080920.4.0000.0068-A-05-06-20; approval date: July 24, 2020). Informed consent was obtained from all participants.

### Participants

All medical students enrolled in the FMUSP from the first to the sixth year were contacted via institutional email (medical school in Brazil is a 6-year degree program). Contact was conducted through eight emails sent twice a week. Messages inviting students to participate were also sent to messaging app groups. The FMUSP accepts 175 new students annually, resulting to a final population of 1,050 contacted participants.

### Data collection: Instruments

Socioeconomic data collected included variables such as age, gender, current school year, marital status, religion, family income, self-evaluated academic performance (i.e., good/average/lacking), household status (i.e., living alone/with parents or grandparents/with friends), and student academic results (i.e., passed, failed, leave of absence, or dismissal).

The use of alcohol and other drugs was assessed using a questionnaire used in previous studies conducted by our group,^21,22^ which evaluated substance use during lifetime and in the previous 12 months. Consumption was assessed by the question “Have you used—at any time in your life or in the last 12 months—[name of the drug] without medical guidance?”.

The following substances were surveyed: alcohol; tobacco; marijuana (and hashish); hallucinogens (LSD, mushroom tea, and mescaline); cocaine; crack cocaine; amphetamines; anticholinergics; benzodiazepines; opiates; sedatives and barbiturates; anabolic steroids; and inhalants.

The impact of the pandemic on drug use was assessed among students who reported using any substance within the last 12 months. Missing values of the remaining students were filled with “Didn't use.” Frequency of use was assessed before and during the pandemic with the following question: “How frequently did you use [name of the drug] before the pandemic started?” and “How frequently do you use [name of drug] now?”. Consumption patterns before and after the COVID-19 outbreak were categorized as (1) less than once a week or (2) once a week or more.

Symptoms of depression were assessed using the Patient Health Questionnaire-9 (PHQ-9),^23^ a 9-item depression module from the full PHQ, with an extra final question asking, “How difficult have these problems made it for you to do your work, take care of things at home, or get along with other people?”. This self-administered scale ranges from 0 to 27, as each of the 9 items can be scored from 0 (not at all) to 3 (nearly every day).

### Analysis

The minimum sample size for a 95% confidence level and a 5% level of precision was obtained using Cochran's formula for sample size calculation, adjusted by finite population correction. The degree of variability was obtained from a previous study conducted by our group, which evaluated the use of alcohol and other drugs by medical students from the University of São Paulo Medical School in 2001.^22^ Using 24.3% as the previous rate of lifetime marijuana use among students, the minimum sample size was estimated at 225 students.

Collected variables were compared between genders and according to the academic year (grouped into the basic cycle, composed of first and second years; clinical cycle, composed of third and fourth years; and internship, composed of fifth and sixth years) using t-tests or χ^2^ tests, according to variable type. Frequency of substance use was compared using χ^2^ tests. The association between substance use and socioeconomic factors was evaluated using linear models, with substance use as the dependent variable and family income, household status, self-evaluated academic performance, and academic results as independent variables, controlling for gender and age. The association between tobacco use and mental health symptoms was also investigated using linear models, with tobacco use as the dependent variable and mental health scores as independent variables, while controlling for gender, age, household income, and current school year. All analyses were performed in Python 3.7.6 (Python Software Foundation, Wilmington, United States)^24^ using the statsmodels package.^25^ Statistical significance was set at an alpha of 0.05.

## RESULTS

### Participants

From 1,050 contacted students, data were collected from 275 participants who completed the questionnaires. The sample comprised 159 (57.8%) males, 113 (41.1%) females, and 3 (1.1%) non-binary gender students. The mean age was 23 years old, and most students were single, with no religious affiliations, and from affluent classes. Seventy-eight (28.4%) students were in the basic cycle, 111 (40.4%) in the clinical cycle, and 86 (31.3%) in internship (Table 1). Non-binary gender students were excluded from some comparisons because of the small sample size.

**Table 1:** Demographics characteristics of enrolled participants

n = 275		
**Academic cycle, n (%)**		
	Basic	78 (28.4)
	Clinical	111 (40.4)
	Internship	86 (31.3)
**Gender, n (%)**		
	Male	159 (57.8)
	Female	113 (41.1)
	Non-binary	3 (1.1)
	Age, mean (SD)	23.3 (3.6)
**Marital status, n (%)**		
	Married	7 (2.5)
	Single	268 (97.5)
**Religion, n (%)**		
	No	174 (63.3)
	Yes	101 (36.7)
**Family income, n (%)**		
	More than 20 MW	73 (26.5)
	10-20x MW	66 (24.0)
	4-10x MW	78 (28.4)
	2-4x MW	47 (17.1)
	0-2 MW	11 (4.0)

SD = standard deviation; MW = minimum wage.

### Substance use among medical students

Regarding lifetime use, alcohol was the most consumed substance in our sample (95.6%, n = 263), followed by marijuana (60%, n = 165), and tobacco (57.5%, n = 158). Additionally, 168 (61.1%) students reported consuming illicit drugs. Substances that were most consumed in the last year were alcohol (82.9%, n = 228), marijuana (42.5%, n = 117), and tobacco (36%, n = 99). Moreover, 123 (44.7%) students reported using illicit drugs. The general prevalence of all drugs is shown in Table 2.

**Table 2:** Last year and lifetime prevalence of substance use among medical students

Substance	Lifetime	Last year
Alcohol	263 (95.6)	228 (82.9)
Marijuana	165 (60.0)	117 (42.5)
Tobacco	158 (57.5)	99 (36.0)
Ecstasy	87 (31.6)	66 (24.0)
Hallucinogens	76 (27.6)	53 (19.3)
Inhalants	60 (21.8)	30 (10.9)
Amphetamines	55 (20.0)	32 (11.6)
Tranquilizers	35 (12.7)	23 (8.4)
Cocaine	34 (12.4)	21 (7.6)
Opiates	18 (6.5)	8 (2.9)
Steroids	5 (1.8)	2 (0.7)
Sedatives	3 (1.1)	1 (0.4)
Crack cocaine	2 (0.7)	0 (0.0)
Anticholinergics	1 (0.4)	0 (0.0)

### Factors associated with substance use

Considering the previous 12-month period, students with a higher family income (more than 20 minimum wages) had higher prevalence rates of substance use than students with a lower family income (less than two minimum wages) (P < 0.01). During assessment, the Brazilian minimum wage was approximately USD 236. The same association was observed in the last year of alcohol use group (P < 0.01). Regarding the last year of tobacco use, we found that living with family members was a protective factor compared to living with friends (P = 0.04). Tobacco use was associated with worse self-evaluated academic performance, with a higher prevalence rate of tobacco use among students who evaluated their academic performance as ‘average/lacking’ compared to students who rated their academic performance as ‘good’ (P = 0.04). Analyzing mental health data with tobacco consumption, we found that students who used tobacco in the previous year had more severe depression symptoms, as measured by the PHQ-9 scale (P = 0.01). Last year, using any illegal substance, living with family members (P < 0.01), or alone (P = 0.02) was a protective factor compared to living with friends, and was also associated with worse self-evaluated academic performance (P = 0.04).

Analysis of academic results of the students (passed, failed, leave of absence, and dismissal) did not show any association with substance use.

### COVID-19 pandemic impact on drug use

Significant differences were observed in the frequency of substance use before and after the COVID-19 outbreak for alcohol, tobacco, marijuana, cocaine, inhalants, hallucinogens, and ecstasy (Table 3). In general, there was a decrease in the frequency of use when the pandemic started, with a reduction in the number of students who reported using less than once a week (alcohol: pre-pandemic 135 versus 107 during pandemic, P < 0.01; tobacco: 52 versus 17, P < 0.01; cannabis: 83 versus 36, P < 0.01; cocaine: 20 versus 2, P < 0.01; inhalants: 29 versus 4, P < 0.01; hallucinogens: 50 versus 24, P < 0.01; ecstasy: 64 versus 9, P < 0.01) and an increase in the number of students who reported not using at all (alcohol: pre-pandemic 48 versus 98 during the pandemic, P < 0.01; tobacco: 186 versus 225, P < 0.01; cannabis: 163 versus 209, P < 0.01; cocaine: 255 versus 270, P < 0.01; inhalants: 246 versus 271, P < 0.01; hallucinogens: 225 versus 251, P < 0.01; ecstasy: 210 versus 265, P < 0.01). However, the number of students who reported using these substances more than once a week remained stable during this period.

**Table 3:** Frequencies of substance use among medical students before and after the coronavirus disease 2019 pandemic outbreak

Frequencies				
		Before	After	P value
Alcohol	Didn't use	48	98	**< 0.01**
	< once/week	135	107	
	≥ once/week	92	70	
Tobacco	Didn't use	186	225	**< 0.01**
	< once/week	52	17	
	≥ once/week	37	33	
Marijuana	Didn't use	163	209	**< 0.01**
	< once/week	83	36	
	≥ once/week	29	30	
Cocaine	Didn't use	255	270	**< 0.01**
	< once/week	20	2	
	≥ once/week	0	3	
Crack	Didn't use	275	275	-
	< once/week	0	0	
	≥ once/week	0	0	
Amphetamines	Didn't use	245	257	0.18
	< once/week	20	13	
	≥ once/week	10	5	
Anticholinergics	Didn't use	275	275	-
	< once/week	0	0	
	≥ once/week	0	0	
Tranquilizers	Didn't use	255	262	0.13
	< once/week	16	7	
	≥ once/week	4	6	
Opiates	Didn't use	267	273	0.11
	< once/week	7	2	
	≥ once/week	1	0	
Sedatives	Didn't use	274	274	1
	< once/week	0	1	
	≥ once/week	1	0	
Steroids	Didn't use	273	275	0.5
	< once/week	2	0	
	≥ once/week	0	0	
Inhalants	Didn't use	246	271	**< 0.01**
	< once/week	29	4	
	≥ once/week	0	0	
Hallucinogens	Didn't use	225	251	**< 0.01**
	< once/week	50	24	
	≥ once/week	0	0	
Ecstasy	Didn't use	210	265	**< 0.01**
	< once/week	64	9	
	≥ once/week	1	1	

Significant comparisons are indicated in bold.

### Drug use according to gender and academic year

In lifetime assessments, male students consumed more ecstasy (males 37.1% versus females 23.9%, P = 0.029) and hallucinogens (males 32.7% versus females 20.4%, P = 0.035). In the last 12 months, males also consumed more tobacco (males 40.9% versus females 28.3%, P = 0.045) and ecstasy (males 28.9% versus females 16.8%, P = 0.03) (Table 4). For all other surveyed substances, there were no significant differences between genders.

**Table 4:** Last year and lifetime prevalence of substance use among medical students, by gender

		Grouped by gender		
		Male	Female	P value
Lifetime	Alcohol	151 (95.0)	109 (96.5)	0.766
	Tobacco	94 (59.1)	62 (54.9)	0.566
	Marijuana	93 (58.5)	70 (61.9)	0.654
	Cocaine	23 (14.5)	9 (8.0)	0.147
	Crack	2 (1.3)	0 (0.0)	0.513
	Amphetamines	37 (23.3)	17 (15.0)	0.128
	Anticholinergics	0 (0.0)	0 (0.0)	-
	Tranquilizers	18 (11.3)	16 (14.2)	0.609
	Opiates	9 (5.7)	7 (6.2)	1
	Sedatives	1 (0.6)	2 (1.8)	0.572
	Steroids	2 (1.3)	2 (1.8)	1
	Inhalants	40 (25.2)	19 (16.8)	0.135
	Hallucinogens	52 (32.7)	23 (20.4)	**0.035**
	Ecstasy	59 (37.1)	27 (23.9)	**0.029**
Last year	Alcohol	130 (81.8)	95 (84.1)	0.739
	Tobacco	65 (40.9)	32 (28.3)	**0.045**
	Marijuana	74 (46.5)	41 (36.3)	0.118
	Cocaine	13 (8.2)	7 (6.2)	0.703
	Crack	0 (0.0)	0 (0.0)	-
	Amphetamines	22 (13.8)	9 (8.0)	0.191
	Anticholinergics	0 (0.0)	0 (0.0)	-
	Tranquilizers	10 (6.3)	12 (10.6)	0.287
	Opiates	2 (1.3)	5 (4.4)	0.131
	Sedatives	0 (0.0)	1 (0.9)	0.415
	Steroids	1 (0.6)	1 (0.9)	1
	Inhalants	22 (13.8)	7 (6.2)	0.07
	Hallucinogens	37 (23.3)	15 (13.3)	0.056
	Ecstasy	46 (28.9)	19 (16.8)	**0.03**

Significant comparisons are indicated in bold.

When assessing differences between medical school years, there was a significant difference between the basic cycle and the latter years. Significant difference between groups was observed for lifetime use of marijuana (basic 41% versus clinical 64.9% versus internship 70.9%, P < 0.001), cocaine (basic 5.1% versus clinical 12.6% versus internship 18.6%, P = 0.028), amphetamines (basic 10.3% versus clinical 21.6% versus internship 26.7%, P = 0.027), inhalants (basic 10.3% versus clinical 23.4% versus internship 30.2%, P = 0.007), hallucinogens (basic 11.5% versus clinical 33.3% versus internship 34.9%, P = 0.001) and ecstasy (basic 19.2% versus clinical 35.1% versus internship 38.4%, P = 0.018). Additionally, significant differences between the groups were observed for last year use of marijuana (basic 26.9% versus clinical 53.2% versus internship 43%, P = 0.002), cocaine (basic 2.6% versus clinical 7.2% versus internship 12.8%, P = 0.045), hallucinogens (basic 7.7% versus clinical 25.2% versus internship 22.1%, P = 0.008), and ecstasy (basic 12.8% versus clinical 27.9% versus internship 29.1%, P = 0.024) (Table 5).

**Table 5:** Last year and lifetime prevalence of substance use among medical students, by academic cycle

		Grouped by Academic cycle				Post-hoc comparisons		
						Basic - Clinical	Basic - Internship	Clinical - Internship
		Basic	Clinical	Internship	P value	P value*	P value*	P value*
Lifetime	Alcohol	72 (92.3)	108 (97.3)	83 (96.5)	0.291	-	-	-
	Tobacco	38 (48.7)	66 (59.5)	54 (62.8)	0.164	-	-	-
	Marijuana	32 (41.0)	72 (64.9)	61 (70.9)	< 0.001	**< 0.001**	**0.004**	0.957
	Cocaine	4 (5.1)	14 (12.6)	16 (18.6)	0.028	0.085	**0.031**	1.000
	Crack	1 (1.3)	0 (0.0)	1 (1.2)	0.517	-	-	-
	Amphetamines	8 (10.3)	24 (21.6)	23 (26.7)	0.027	**0.017**	**0.026**	1.000
	Anticholinergics	0 (0.0)	0 (0.0)	1 (1.2)	0.596	-	-	-
	Tranquilizers	7 (9.0)	16 (14.4)	12 (14.0)	0.499	-	-	-
	Opiates	2 (2.6)	7 (6.3)	9 (10.5)	0.121	-	-	-
	Sedatives	2 (2.6)	1 (0.9)	0 (0.0)	0.372	-	-	-
	Steroids	1 (1.3)	1 (0.9)	3 (3.5)	0.523	-	-	-
	Inhalants	8 (10.3)	26 (23.4)	26 (30.2)	0.007	**0.006**	**0.006**	1.000
	Hallucinogens	9 (11.5)	37 (33.3)	30 (34.9)	0.001	**< 0.001**	**0.002**	1.000
	Ecstasy	15 (19.2)	39 (35.1)	33 (38.4)	0.018	**0.003**	**0.029**	1.000
Previous year	Alcohol	59 (75.6)	95 (85.6)	74 (86.0)	0.131	-	-	-
	Tobacco	22 (28.2)	48 (43.2)	29 (33.7)	0.092	-	-	-
	Marijuana	21 (26.9)	59 (53.2)	37 (43.0)	0.002	**< 0.001**	0.110	0.054
	Cocaine	2 (2.6)	8 (7.2)	11 (12.8)	0.045	0.318	0.063	1.000
	Crack	78 (100.0)	111 (100.0)	86 (100.0)	NA	-	-	-
	Amphetamines	4 (5.1)	15 (13.5)	13 (15.1)	0.086	-	-	-
	Anticholinergics	78 (100.0)	111 (100.0)	86 (100.0)	NA	-	-	-
	Tranquilizers	4 (5.1)	11 (9.9)	8 (9.3)	0.495	-	-	-
	Opiates	0 (0.0)	3 (2.7)	5 (5.8)	0.066	-	-	-
	Sedatives	0 (0.0)	1 (0.9)	0 (0.0)	1	-	-	-
	Steroids	0 (0.0)	0 (0.0)	2 (2.3)	0.177	-	-	-
	Inhalants	5 (6.4)	12 (10.8)	13 (15.1)	0.203	-	-	-
	Hallucinogens	6 (7.7)	28 (25.2)	19 (22.1)	**0.008**	**< 0.001**	**0.038**	0.666
	Ecstasy	10 (12.8)	31 (27.9)	25 (29.1)	**0.024**	**0.003**	**0.040**	1.000

NA = not applicable.

* P value: Bonferroni-adjusted P value. Basic: first and second year; Clinical: third and fourth year; Internship: fifth and sixth year. Significant comparisons are indicated in bold.

Post-hoc analyses comparing individual groups revealed that drug use prevalence mainly differed between basic and clinical cycles. Significant differences, after Bonferroni correction for multiple comparisons between these two groups, were observed for lifetime use of hallucinogens (p_adj_ < 0.001), amphetamines (p_adj_ = 0.017), inhalants (p_adj_ = 0.006), marijuana (p_adj_ < 0.001), and ecstasy (p_adj_ = 0.003), and for use in the last year of hallucinogens (p_adj_ < 0.001), marijuana (p_adj_ < 0.001), and ecstasy (p_adj_ = 0.003). The use of cocaine differed only in terms of lifetime use between basic and internship cycles (p_adj_ = 0.031). In contrast, using for the last year did not differ between groups after correction (Table 5).

## DISCUSSION

This study evaluated the prevalence of substance use among students from a medical school in São Paulo, Brazil, during the COVID-19 pandemic. After the COVID-19 outbreak, we observed decreased use among students who reported less than once weekly use of substances and maintained use among students who reported weekly drug use. Finally, we compared the consumption rates between genders and academic years to identify which students were more prone to substance use.

The rate of substance use in our sample was higher than that among the general population of the same age^26^ and general university students in Brazil.^27^ Notably, cannabis is now the second most used drug among medical students, consistent with the more general trend observed among high school students in the United States^28^ and elsewhere. Additionally, the way cannabis is seen or used among medical students may influence the detection of substance use problems among patients in their future medical practice, as physician experience with drugs may affect how they perceive these problems.^29^ Physicians may also participate in developing future drug policies, and their attitudes as well as experiences with substances may influence their roles.

Tobacco use has almost doubled in the last 19 years when current data were compared to those obtained in the same sample in 2001.^22^ This was unexpected as Brazil has a very efficient program to control smoking,^30^ with national rates continuously decreasing. Still, although other studies did not observe a similar increase,^31^ consumption rate was similar to that observed among general university students in Brazil.^32^ Alternative tobacco products (i.e., little cigars and cigarillos, e-cigarettes, and hookah), with potential appeal to the youth, may be associated with increased tobacco consumption among our population.^33^ Moreover, data showed that tobacco users in the last year had worse self-evaluated academic performance, and more severe depression symptoms measured by the PHQ-9 scale. The literature demonstrates a significant association between smoking and depression risk^34^ as well as symptoms.^35^ Additionally, a study conducted among Canadian medical students showed an association between current tobacco use and higher psychological distress. This study suggests that substance use may be a coping mechanism to deal with stress and the risk of burnout.^36^ This may also be the case of students from our sample, and tobacco may serve as a proxy to detect psychologically vulnerable students.

Ecstasy and hallucinogen use in this study was much higher than that among the general population and other medical students;^26,37^ although there is a general trend observed in other countries of increasing use of those substances.^38–41^ In our sample, during last year, cocaine was used by 7.6% of the students, a higher rate than that from previous studies investigating Brazilian medical students.^37,42^ Finally, amphetamine use was more frequent in our sample than in other studies, whereas alcohol and inhalant consumption rates were similar.^32,37,43,44^

Regarding the impact of the COVID-19 outbreak on the frequency of substance use, our findings suggest an overall reduction in consumption. There was a decrease in the number of students who reported “less than once a week” use of alcohol, tobacco, cannabis, cocaine, inhalants, hallucinogens, and ecstasy. On the other hand, the frequency of students who used drugs “once or more than once a week” remained constant during the COVID-19 pandemic. Social situations, such as parties where exposure to these substances occur, stopped happening after the pandemic started, which may have led to a decrease in substance use among infrequent users. In contrast, for higher-frequency users, our findings did not show a significant difference after the advent of the pandemic. This might be due to these students making use of these substances irrespective of social gatherings. Other studies evaluating the effects of the pandemic on substance use have reported similar findings, with a decrease in the overall consumption prevalence but an increase in the frequency of marijuana, alcohol, and tobacco use among users.^19,45^

We also analyzed the influence of gender and academic year on drug consumption. In our sample, men consumed more tobacco, ecstasy, and hallucinogens than women did. These findings are in line with other studies analyzing Brazilian and American populations.^26,46^ Regarding alcohol consumption among medical students, we found convergent use between genders, which was also observed in other non-medical student samples.^22,47–49^

A study conducted in 2001 in our setting reported greater use of amphetamines by women,^22^ but this difference seems to be diminishing. At that time, amphetamines were mainly anorectic drugs (amfepramone, mazindol, and phenylethylamine) used by young women to reduce weight. These drugs were proscribed in Brazil in 2011. The currently available amphetamines in Brazil are mainly used for treating attention deficit hyperactivity disorder. Currently, non-medical use of amphetamines is primarily for academic performance improvement (“academic doping”), an increasingly common behavior among both genders, contributing to reducing the differences seen in previous studies.^50–52^

This study also showed a significant increase in the use of hallucinogens, amphetamines, cocaine, inhalants, marijuana, and ecstasy from the basic to clinical cycle. This is not surprising, given the age differences between cycles, and may reflect the natural progression of growing older and being exposed to novel experiences.^22,44^ However, besides aging, this finding could also be attributed to the increasing academic, professional, and financial stressors as medical students progress to their final clinical years.^53,54^ Therefore, medical school managers must be aware of this trend and implement preventive actions to avoid or mitigate it.

As medical schools are frequently linked to hospital settings, students should have ready access to mental healthcare. Unfortunately, the literature does not confirm this assumption.^55,56^ Moreover, in the medical student milieu, substance use policies are often punitive and stigmatized.^57^ Asking for emotional help can be seen as a weakness and career-threatening.^58^ Therefore, mental health care should be actively offered to this population.

This study had some limitations. First, our estimates were assessed in the context of the COVID-19 pandemic, following the implementation of social distancing measures. The effects of this confounder could not be discriminated within our sample, potentially influencing the drug use profile for the last year. However, the overall data shown in this study are compatible with those of other studies that evaluated substance use among medical students and studies conducted in the COVID-19 pandemic context. Second, participant responses may be subject to memory bias, which is a prevalent concern in retrospective studies. To minimize this effect, we used a standardized questionnaire to assess substance use during specific and objective periods. Additionally, despite being higher than the sample size estimates, our response rate (26.2 %) can be considered low and may reduce external validity. However, the distribution of respondents according to gender and academic cycle is consistent with the general proportion of students in our institution. Therefore, it was difficult to determine how the relatively low response rate affected the results. Moreover, our study is based on a convenience sample that may impair the representativeness of our results. This limitation is common in surveys for this type of population and has been observed in most studies on this subject.^2,31,40,50,51^

## CONCLUSION

This study showed a high prevalence of substance use among medical school students. There was a trend toward convergent substance consumption between genders, and drug use increased from the basic to the clinical cycle. The COVID-19 pandemic may have influenced prevalence estimates, decreased the frequency of substance use among sporadic users, but it did not significantly affect frequent users. Drug use among this population may have important impacts on student mental health at present, and on patient care as well as health policies in the future.
